# Genetic Diversity and Haplotype Analysis of Cattle Hydatid Cyst Isolates Using Mitochondrial Markers in Turkey

**DOI:** 10.3390/pathogens11050519

**Published:** 2022-04-28

**Authors:** Harun Kaya Kesik, Figen Celik, Seyma Gunyakti Kilinc, Sami Simsek, Haroon Ahmed, Yujuan Shen, Jianping Cao

**Affiliations:** 1 Department of Parasitology, Faculty of Veterinary Medicine, University of Bingöl, Bingöl 12300, Turkey; hkesik@bingol.edu.tr (H.K.K.); sgunyakti@bingol.edu.tr (S.G.K.); 2 Department of Parasitology, Faculty of Veterinary Medicine, University of Firat, Elazig 23119, Turkey; f.celik@firat.edu.tr; 3 Department of Biosciences, COMSATS University Islamabad (CUI), Park Road, Chak Shahzad, Islamabad 45550, Pakistan; haroonahmed@comsats.edu.pk; 4 Key Laboratory of Parasite and Vector Biology, National Health Commission of People’s Republic of China, Shanghai 200025, China; shenyj@nipd.chinacdc.cn; 5 National Institute of Parasitic Diseases, Chinese Center for Disease Control and Prevention, Chinese Center for Tropical Diseases Research, Shanghai 200025, China; 6 The School of Global Health, Chinese Center for Tropical Diseases Research, Shanghai Jiao Tong University School of Medicine, Shanghai 200025, China; 7 WHO Collaborating Centre for Tropical Diseases, Shanghai 200025, China

**Keywords:** hydatid disease, *Echinococcus granulosus sensu stricto*, cattle, haplotype, Turkey

## Abstract

*Echinococcus granulosus sensu lato* (s.l.) causes cystic echinococcosis in ungulates and humans. The current study was designed to find the genetic diversity and haplotypic profiles of hydatid cysts from the lungs of cattle in three provinces in eastern Turkey. Individual cyst isolates (n = 60) were collected from infected cattle lungs after slaughter and then samples were stored in ethanol (70%) until further use. From each isolate, total gDNA was extracted from the cysts’ germinal layers. A partial (875 bp) *mt*-*CO1* gene was amplified by PCR and sequenced unidirectionally. The final size of the trimmed sequences was 530 bp for 60 sequences. Sequence and haplotype analyses were performed, followed by phylogenetic analyses. According to BLAST searches, all sequences were detected as *E. granulosus s.s.* (G1 and G3 strains). Forty-nine point mutations were identified. In addition, five conserved fragments were detected in all sequences. The haplotype analysis diagram showed *E. granulosus s.s*. haplotypes organized in a star-like configuration. The haplotypes were characterized by 1–17 mutations compared with the fundamental focal haplotype. Thirty-three haplotypes were determined in 60 samples of which 17 (28.3%) belonged to the main haplotype (Hap_06). The *mt*-*CO1* sequences revealed 49 polymorphic sites, 34.5% (20/49) of which were informative according to parsimony analysis.

## 1. Introduction

*Echinococcus granulosus sensu lato*, a species complex, is responsible for cystic echinococcosis (CE) in many ungulates and humans [1]. Globally, CE is one of the most serious infectious disease because of its significant financial burden and public health implications [2]. Animals such as goats, sheep, camels, pigs, and cattle act as intermediate hosts, harboring the metacestodes in hydatid cysts that infect the liver, lungs, and other parts of the body. Moreover, the adult tapeworm resides in the intestines of definitive hosts, which are canids including domestic dogs [3].

Genetic diversity of *E. granulosus s.l.* complex were investigated from many species of intermediate hosts in the last five decades. [4]. Molecular characterization of the *E. granulosus s.l.* complex has been achieved: *E. granulosus sensu stricto* (*s.s*) (G1 and G3 strains), *E. equinus* (G4), *E. ortleppi* (G5), *E. canadesis* (G6–G10), and *E. felidis* (lion strain) [5]. 

In various areas of the world, CE is highly endemic and widespread, especially in rural settings in most parts of the world, such as Africa, the Middle East, Mediterranean Europe, South America, Central Asia, and Western China, e.g., rural and pastoral communities where humans and animals live in close association with each other [6]. CE is also prevalent in almost every region of Turkey [7]. In recent years, several studies reported the prevalence of CE in livestock from Turkey. The overall recorded prevalence ranged from 3.5 to 58.6%. The prevalence was determined according to the results of studies for various species of animals ranging from 3.5 to 70.9% in sheep, 1.6 to 25.1% in goats, 3 to 46.4% in cattle, and 10.2 to 41.1% in buffaloes [8,9,10,11,12,13]. CE was responsible for a total yield loss of 89.2 million USD for animals (cattle, sheep, and goats) in 2008 in Turkey [14].

In recent molecular studies, three species, *E. granulosus s.s*., *E. eqinus*, and *E. canadensis* (G6/G7), were observed to infect intermediate hosts, and *E. granulosus s.s.* was reported as a predominant species among cattle in Turkey [11,15,16].

The present study hypothesized that the *mt*-*CO*1 sequence could be used to genetically identify cattle isolates of hydatid cysts and could determine the haplotype profile of *E. granulosus s.l.* in three provinces of Turkey. 

## 2. Results

We collected 60 samples of cattle hydatid cysts. All of the isolates were obtained from lungs. To determine the exact species of the isolates, PCR was performed to amplify a *mt*-*CO*1 gene fragment, and in all samples an 875 bp band was amplified.

The nucleotide sequences were compared with published reference sequences and trimmed. The final size of the trimmed sequences was 530 bp for 60 sequences (BNG41-BNG100). All sequences were identified as *E. granulosus s.s.* (G1 and G3), according to BLAST searches. All the sequences were then submitted to GenBank and accession numbers (MW020975-MW021034) were obtained, belonging to all nucleotide sequences.

After the alignment, point mutations in the sequences were identified. Nucleotide variation positions based on the reference sequence are shown in Table 1. The accession numbers of *E. granulosus s.s.* (G1 and G3) (AY389989, KT001408, MG808348), *E. canadensis* (G6/G7) (KX010870, KX010869, LC184604, LC184606), *E, ortleppi* (G5) (KU743926, MN886291), and *E. multilocularis* (AB510025) were used for phylogenetic tree construction and the *Taenia saginata* (KY290361) sequence was used as an outgroup. 

**Figure 1:**
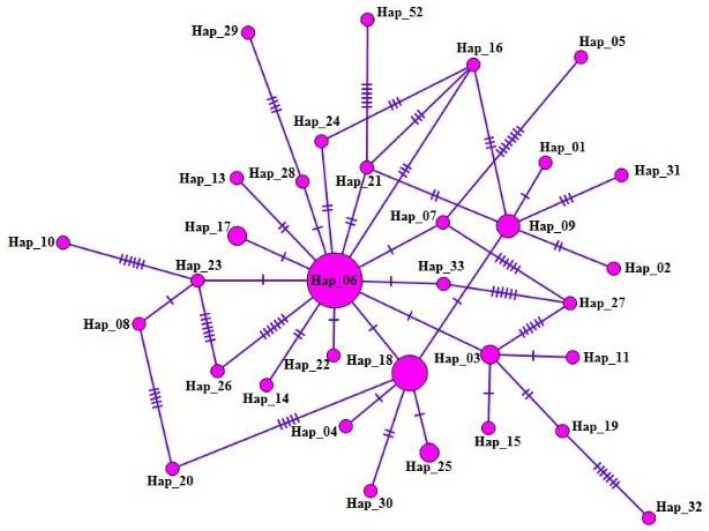
The haplotype network for the *mt-CO1* (530 bp) gene of *E. granulosus s.s.* The size of the circles is proportional to the frequency of each haplotype. The number of mutations separating haplotypes is indicated by dash marks. Hap: haplotype.

As a result of haplotype analyses and subsequent to essential focal haplotype, *E. granulosus s.s.* was configured in orientation like a star. 1–17 mutation steps segregated it from other haplotypes; 33 haplotypes were determined in 60 samples, of which 17 (28.3%) belonged to the main haplotype (Hap_06) (Figure 1; Table 2). In the *mt-CO1* gene sequences, 49 polymorphic sites were identified by performing the alignment with the reference sequence (NC044548; Table 1), among which 34.5% (20/49) were informative according to parsimony analysis. In addition, five conserved regions (7–39, 41–111, 181–270, 272–301, and 308–354 bp) were identified in all the sequences. Nine haplotypes (italic ones in Table 2) detected in this study were 100% similar to the previously published reference sequences in the Genbank while 24 haplotypes were unique and were revealed for the first time in this study.

In current report, higher haplotype variation and lower levels of nucleotide differences were detected for the *mt-CO1* gene (Table 3). Population buildup and/or refinement of selection was indicated by Tajima’s D value. Uncommon haplotypes from recent population expansion or hitchhiking were expected because of the significant negative Fu’s Fs value. Hence, the fact that 81.8% (27/33) of the haplotype groups were single haplotypes supported these results.

## 3. Discussion

Cystic echinococcosis, an endemic zoonotic disease acquired by cestode parasite of *E. granulosus s.l.* It is a neglected tropical disease according to the World Health Organization (WHO), causing serious medical and economic losses every year [17]. PCR- based methods are frequently used to identify and genotype species/strains within the *E. granulosus s.l.* complex.

Determining the prevalent strains in an endemic region such as Turkey is highly significant for investigating its pathogenicity, pattern of transmission, parasite life cycle, CE diagnosis and treatment methods, possible eradication, vaccine and drug studies, and parasite control. For the genotypic and molecular identification of *E. granulosus s.l.* nuclear and mitochondrial genes have been utilized. Phylogenetic analysis of closely related species, mitochondrial genes are preferred over nuclear genes because of their haploid properties, rapid sequence evolution, and large datasets [18]. 

*E. granulosus s.l.* comprises different genotypes and is further divided into *E. granulosus s.s.*, *E. ortleppi*, *E. equinus*, *E. canadensis* (G6/G7, G8 and G10), and *E. felidis* [5]. *E. granulosus s.s.* is the most eminent species in Turkey [19]. *E. granulosus s.s.* generally has an extensive distribution in China [20], some parts of Europe [21,22,23], South America [24,25], and Africa [26] among all intermediate hosts and humans. Therefore, it is not alarming that in Turkey *E. granulosus s.s.* is a common species. Pigs and camels, the intermediate hosts of *E. canadensis* (G6/G7), are at a minimum level in Turkey. The main source of income in Turkey is the breeding of cattle and sheep, which have a high prevalence of *E. granulosus s.s*. One of the main aims of the present study was to investigate the possible presence of *E. ortleppi* in CE-infected cattle lungs, because *E. ortleppi* in stray dogs was reported recently in Turkey [27]. However, in this study, in all 60 samples, only *E. granulosus s.s.* was identified among cattle lungs infected with hydatid cysts.

A recent study from Turkey reported that 39 out of the 40 cattle hydatid cyst isolates were detected as *E. granulosus s.s.* However, one cattle isolate matched with *E. canadensis* (G6/G7) according to the *mt-CO1* gene sequences (758 bp) [28]. Another study was performed on 120 isolates, 114 of which showed CE infection by *E. granulosus s.s.* using 12S rRNA PCR, while the remaining 6 isolates showed *E. granulosus s.s.* infection after *mt*-*CO1* gene analysis [16]. According to previous reports performed on cattle isolates (n = 57), *E. granulosus s.s.* was determined after PCR and sequence analysis in Konya province of Turkey [29]. In addition, among 85 sheep and cattle isolates from Turkey (Elazig province), 84 hydatid cyst was characterized as *E. granulosus s.s.* as a result of sequence analysis [30]. All these conducted investigations reported that *E. granulosus s.s.* was the eminent species reported in cyst samples from different areas of Turkey. In the present study, 60 cattle isolates were confirmed as *E. granulosus s.s.* in three provinces of Turkey using *mt*-*CO1* gene sequencing and the sequences showed similarity with reference sequences of *E. granulosus s.s.*

We determined 33 *E. granulosus s.s.* haplotypes among the 60 cattle isolates and also observed multiple nucleotide mutation positions (49 point mutations) within these 33 haplotypes. The identification of 33 haplotypes in 60 cattle isolates proved the significant genetic variability of *E. granulosus s.s.* A recent study indicated relatively high G1 strain haplotype diversity, because 171 haplotypes in 212 samples were determined in different regions of the world [19]. According to the haplotype analysis for the *mt*-*CO1* gene, high haplotype diversity and low nucleotide variation were noted. Tajima’s D value indicated expansion of the population and the influence of purifying selection. The negative value of Fu’s Fs indicated the presence of rare haplotypes occurred because of hitchhiking or recent population enlargement. Hence, it was not surprising that there were 33 haplotypes out of 60 sequences. Indeed, 81.8% of the haplotype comprised a single sequence.

## 4. Material and Methods

### 4.1. Study Area and Sample Collection

**Figure 2:**
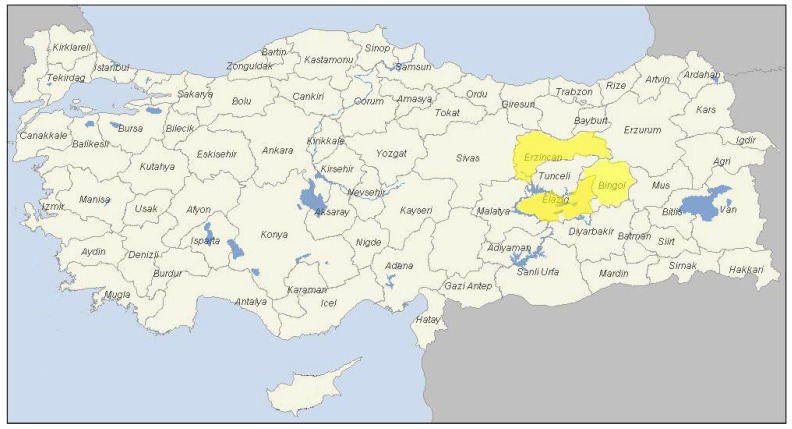
The provinces (in yellow) from which the hydatid cyst samples were collected from Turkey.

A total of sixty (n = 60) cattle hydatid cysts were gathered from slaughterhouses in Bingol, Elazig, and Erzincan provinces of eastern Turkey from October 2019 to February 2020 (Figure 2). Following slaughter, the internal organs of each animal, especially the lungs, were examined by both inspection and palpation for the presence of hydatid cysts. Only one hydatid cyst sample was taken from each cattle. Germinal membranes were separated from the detected hydatid cysts using scissors, placed into 70% ethanol, and delivered to the laboratory on ice. Each collected cyst was stored at −20 °C in 70% ethanol in separate Eppendorf tubes for subsequent experiments. 

### 4.2. Genomic DNA Isolation from Individual Cyst Samples

The protocol, as prescribed by the manufacturer, was performed for genomic DNA (gDNA) extraction using a Hibrigen Genomic DNA Isolation Kit (Hibrigen, Kocaeli, Turkey), according to the instructions of manufacturers company but with slight adjustments. The tissue samples were then washed five times with phosphate buffered saline (PBS) to remove excess amounts of alcohol and then the germinal layer was dissected into small segments using a scalpel, and then transferred into Eppendorf tubes (1.5 mL). Two hundred microliters of lysis solution and twenty microliters (20 mg/ml) of Proteinase-K were mixed, swirled, and incubated overnight at 65 °C. To obtain effective results, the samples were vortexed every 1 h, at least two to three times. The quantity of gDNA was determined by using a Nanodrop device (Nanodrop Technologies, Wilmington, DE, USA) and those samples containing insufficient gDNA were re-extract to obtain a sufficient amount of gDNA. The extracted gDNA was then kept at −20 °C until further use.

### 4.3. PCR Amplification of mt-CO1 Gene Fragment

The PCR amplification of the *mt*-*CO1* gene (875 bp) fragment was done by using primers F/CO1 forward (5′-TTGAATTTGCCACGTTTGAATGC-3′) and R/CO1 reverse (5′-GAACCTAACGACATAACATAATGA-3′) as previously described [31]. The PCR reaction comprised the following: 5 µL of 10 × PCR buffer; 25 mM of MgCl_2_; each dNTPs (400 µM), primers (each 20 pmol), TaqDNA Polymerase (5 U/µL); PCR grade water (28.8 µL); and 5 µL of gDNA. The cycle parameters of the PCR reaction were as follows: initial denaturation at 94 °C for 10 minutes; 30 cycles of 30 seconds at 94 °C; 45 seconds at 52 °C; and 1 minute at 72 °C; and a final extension step for 10 minutes at 72 °C. A thermal cycler from SensoQuest GmbH (Göttingen, Germany) was used to perform PCR, and 1.4 % agarose gel electrophoresis was used to separate the PCR products.

### 4.4. DNA Sequence Analysis 

PCR products of the *mt-CO1* gene belonging to 60 individual hydatid cysts isolates were sent to the DNA Laboratory of the Genetic Disease Diagnosis Center (Istanbul, Turkey) for unidirectional Sanger DNA sequence. The resulting sequences were compared with GenBank reference sequences using BLAST.

### 4.5. Alignment and Phylogenetic Analysis of mt-CO1 Gene

FinchTV 1.4.0 was utilized to analyze the sequence data. BLAST search was used to compare and trim the sequences. The MEGA X tool was used to evaluate the trimmed sequences [32]. Using NCBI Pubmed published reference sequences as outgroups, alignment of the sequences was performed. The evolutionary tree was generated using the maximum likelihood method in MEGA X, after finding the most accurate and efficient model the best model was used by using the MEGA X program. [32].

### 4.6. Haplotype Networks, Nucleotide Polymorphism, Diversity, and Neutrality Indices 

Following a published protocol [33], all sequence data were uploaded into the DnaSP6 software. The software was used to determine the diversity of population indices and neutrality indices. Population diversity indices included nucleotide diversity (πd), haplotype diversity (hd), and haplotype number (hn), while neutrality indices consisted of Tajima’s D [34], Fu’s statistics [35], Fu’s Fs [21], Fu and Li’s D test (FLD), Fu and Li’s F (FLF) statistics test, and parsimony informative analysis was performed using DnaSP6. Data in the NEXUS output format were generated using DnaSP6 software. To create the haplotype network, the minimum spanning network (MSN) method was used [36] in the PopART-1.7 tool [37].

## 5. Conclusions

In the current study *E. granulosus s.s.* species was identified as the predominant species in cattle hydatid cyst samples in the study area. Among analyzed sequences, the highly prevalent haplotypes were Hap_06 and Hap_18. Nine haplotypes were 100% similar to the published reference sequences while 24 haplotypes were unique. These corresponded to *mt-CO1* haplotypes, which have been detected widely in *E. granulosus s.s.* studies on *mt-CO1* diversity.

## Figures and Tables

**Table 1:** Nucleotide variation positions of the *mt-CO1* (530 bp) gene among 33 analyzed haplotypes. A: Adenine, T: Thymine, C: Cytosine, G: Guanine.

**Nucleotide Positions**	**6**	**40**	**112**		**142**	**143**	**159**	**180**	**271**	**302**	**307**	**355**	**358**	**361**	**377**	**383**	**402**	**422**	**423**	**428**	**430**	**438**	**448**	**458**	**461**
NC_044548 (Reference sequence)	C	C	T		T	C	T	C	C	C	A	C	T	T	A	C	G	A	G	A	T	C	C	T	T
Hap_01	T																					T	T		
Hap_02																						T	T		
Hap_03									T																
Hap_04													C									T			
Hap_05						T																			C
Hap_06																									
Hap_07																									
Hap_08																									
Hap_09																						T	T		
Hap_10																	A								
Hap_11									T						G										
Hap_12												T											T		
Hap_13							C				G														
Hap_14																									
Hap_15									T											G					
Hap_16		T			C																		T		
Hap_17								T																	
Hap_18																						T			
Hap_19									T									G					T		
Hap_20																						T			
Hap_21																							T		
Hap_22																									
Hap_23																									
Hap_24	T				C																				
Hap_25																						T			
Hap_26			C																						
Hap_27									T										A						
Hap_28																									
Hap_29																									
Hap_30																T					A	T			
Hap_31									T													T	T		
Hap_32										A				C			A	G					T	G	
Hap_33																			A						
**Nucleotide Positions**	**479**	**480**	**482**	**484**	**486**	**493**	**497**	**498**	**499**	**500**	**502**	**503**	**504**	**505**	**506**	**507**	**511**	**513**	**515**	**518**	**522**	**523**	**525**	**527**	**528**
NC_044548 (Ref. sequence)	G	T	T	T	T	C	G	T	T	T	G	G	G	T	C	A	T	T	T	A	T	T	G	T	T
Hap_01																									
Hap_02		C																						C	
Hap_03																									
Hap_04																									
Hap_05		A	A			A					C	A	A											C	
Hap_06																									
Hap_07		A																							
Hap_08	C					T																			
Hap_09																									
Hap_10						T			A								A	A				A		A	
Hap_11																									
Hap_12		A	A			A									A								C	A	
Hap_13																									
Hap_14							T																	G	
Hap_15																									
Hap_16																									
Hap_17																									
Hap_18																									
Hap_19																									
Hap_20	C					A		A											A	T					
Hap_21			A																						
Hap_22															T										
Hap_23						T																			
Hap_24																									
Hap_25														C											
Hap_26						A											A	A			A			C	A
Hap_27	C	A		A						A														A	
Hap_28											A														
Hap_29		C			G			A			A								A						
Hap_30																									
Hap_31																C	A								
Hap_32	A																A								
Hap_33																									

**Table 2:** Haplotypes of *mt-CO1* sequences of *E. granulosus s.s.* and accession numbers of isolates forming groups. Those showing 100% similarity to reference sequences are in italics.

No	Haplotype Name	Number of Isolates	Isolate Codes (Accession Numbers)
1	*Hap_01*	1	BNG41(MW020975)
2	Hap_02	1	BNG42(MW020976)
3	*Hap_03*	2	BNG43(MW020977), BNG96(MW021030)
4	Hap_04	1	BNG44(MW020978)
5	Hap_05	1	BNG45(MW020979)
6	*Hap_06*	17	BNG46(MW020980), BNG55(MW020989), BNG59(MW020993), BNG61(MW020995), BNG62(MW020996), BNG73(MW021007), BNG76(MW021010), BNG81(MW021015), BNG84(MW021018), BNG85(MW021019), BNG91(MW021025), BNG93(MW021027), BNG94(MW021028), BNG95(MW021029), BNG97(MW021031), BNG98(MW021032), BNG99(MW021033)
7	Hap_07	1	BNG47 (MW020981)
8	Hap_08	1	BNG48 (MW020982)
9	*Hap_09*	3	BNG49(MW020983), BNG87(MW021021), BNG89(MW021023)
10	Hap_10	1	BNG50(MW020984)
11	Hap_11	1	BNG51(MW020985)
12	Hap_12	1	BNG52(MW020986)
13	*Hap_13*	1	BNG53(MW020987)
14	Hap_14	1	BNG54(MW020988)
15	Hap_15	1	BNG56(MW020990)
16	Hap_16	1	BNG57(MW020991)
17	*Hap_17*	2	BNG58(MW020992), BNG60 (MW020994)
18	*Hap_18*	7	BNG63(MW020997), BNG64(MW020998), BNG66(MW021000), BNG70(MW021004), BNG74(MW021008), BNG82(MW021016), BNG83(MW021017)
19	Hap_19	1	BNG65(MW020999)
20	Hap_20	1	BNG67(MW021001)
21	Hap_21	1	BNG68(MW021002)
22	Hap_22	1	BNG69(MW021003)
23	*Hap_23*	1	BNG71(MW021005)
24	Hap_24	1	BNG72(MW021006)
25	*Hap_25*	2	BNG75(MW021009), BNG100(MW021034)
26	Hap_26	1	BNG77(MW021011)
27	Hap_27	1	BNG78(MW021012)
28	Hap_28	1	BNG79(MW021013)
29	Hap_29	1	BNG80(MW021014)
30	Hap_30	1	BNG86(MW021020)
31	Hap_31	1	BNG88(MW021022)
32	Hap_32	1	BNG90(MW021024)
33	Hap_33	1	BNG92(MW021026)

**Table 3:** Diversity and neutrality indices obtained using nucleotide data of the *E. granulosus s.s*. *mt*-*CO1* gene (530 bp).

mtDNA	n	hn	hd ± SD	πd ± SD	Tajima’s D	*p* Value	Fu’s Fs	*p* Value	FLD	*p* Value	FLF	*p* Value
530 bp	60	33	0.908 ± 0.030	0.00692 ± 0.00101	−2.35657	*p* < 0.01	−27.276	0.0000	−3.51234	*p* < 0.02	−3.67712	*p* < 0.02

n: number of isolates; hn: number of haplotypes; hd: haplotype diversity; πd: nucleotide diversity; SD: standard deviation; FLD: Fu and Li’s D test statistic; FLF: Fu and Li’s F statistics test.

## Data Availability

Not applicable.
